# Efficacy and safety of serplulimab plus nab-paclitaxel in previously treated patients with PD-L1–positive advanced cervical cancer: a phase II, single-arm study

**DOI:** 10.3389/fimmu.2023.1142256

**Published:** 2023-04-21

**Authors:** Jusheng An, Xiumin Li, Jing Wang, Lijing Zhu, Ruifang An, Kui Jiang, Yi Huang, Ke Wang, Guiling Li, Chunyan Wang, Jianlin Yuan, Xiaoli Hou, Guiyu Yang, Jing Li, Qingyu Wang, Jun Zhu, Lingying Wu

**Affiliations:** ^1^ Department of Gynecologic Oncology, National Cancer Center/National Clinical Research Center for Cancer/Cancer Hospital, Chinese Academy of Medical Sciences and Peking Union Medical College, Beijing, China; ^2^ Department of Gynecological Oncology, Linyi Cancer Hospital, Linyi, China; ^3^ Department of Gynecological Oncology, Hunan Cancer Hospital, The Affiliated Cancer Hospital of Xiangya School of Medicine, Central South University, Changsha, China; ^4^ The Comprehensive Cancer Center of Drum Tower Hospital, Medical School of Nanjing University & Clinical Cancer Institute of Nanjing University, Nanjing, China; ^5^ Department of Gynecology and Obstetrics, The First Affiliated Hospital of Xi’an Jiaotong University, Xi’an, China; ^6^ Department of Medical Oncology, The Second Hospital of Dalian Medical University, Dalian, China; ^7^ Department of Gynecological Oncology, Hubei Cancer Hospital, Wuhan, China; ^8^ Department of Gynecological Oncology, Tianjin Medical University Cancer Institute & Hospital, Tianjin, China; ^9^ Department of Oncology, Union Hospital Affiliated to Tongji Medical College of Huazhong University of Science and Technology, Wuhan, China; ^10^ The Fourth Department of Gynecology, Liaoning Cancer Hospital & Institute, Cancer Hospital of China Medical University, Shenyang, China; ^11^ The Third Department of Gynecologic Surgery, Cancer Hospital Affiliated to Xinjiang Medical University, Urumqi, China; ^12^ Global Product Development, Shanghai Henlius Biotech, Inc., Shanghai, China

**Keywords:** serplulimab, PD-1 inhibitor, cervical cancer, phase II, efficacy

## Abstract

**Objective:**

We report the efficacy and safety of serplulimab, a novel humanized anti–programmed death-1 antibody, plus nanoparticle albumin-bound (nab)-paclitaxel in previously treated patients with programmed death ligand-1 (PD-L1)–positive advanced cervical cancer.

**Methods:**

Patients diagnosed with PD-L1–positive (combined positive score ≥1) cervical cancer were enrolled in this single-arm, open-label, phase II study. They were given serplulimab 4.5 mg/kg for up to 2 years (35 dosing cycles) plus nab-paclitaxel 260 mg/m^2^ for up to six cycles once every 3 weeks. Primary endpoints were safety and objective response rate (ORR) assessed by independent radiological review committee (IRRC) per RECIST version 1.1. Secondary endpoints included ORR assessed by the investigator, duration of response (DOR), progression-free survival (PFS), and overall survival (OS).

**Results:**

Between December 2019 and June 2020, 52 patients were screened and 21 were enrolled. IRRC-assessed ORR was 57.1% (95% confidence interval [CI] 34.0–78.2%); 3 (14.3%) patients achieved complete response and 9 (42.9%) partial response. The median DOR was not reached (NR) (95% CI 4.1–NR). IRRC-assessed median PFS was 5.7 months (95% CI 3.0–NR), and median OS was 15.5 months (95% CI 10.5–NR). Investigator-assessed ORR was 47.6% (95% CI 25.7–70.2%). Seventeen (81.0%) patients experienced grade ≥3 treatment-emergent adverse events. Grade ≥3 adverse drug reactions were reported in 7 (33.3%) patients. Immune-related adverse events occurred in 12 (57.1%) patients.

**Conclusions:**

In previously treated patients with PD-L1–positive advanced cervical cancer, serplulimab plus nab-paclitaxel provided durable clinical activity and a manageable safety profile.

**Clinical trial registration:**

ClinicalTrials.gov, identifier NCT04150575.

## 1 Introduction

Cervical cancer is the fourth most prevalent cancer and the fourth leading cause of cancer death in women, with approximately 604,000 new cases and 342,000 cervical cancer-related deaths worldwide in 2020 (1). The incidence and mortality of cervical cancer vary greatly among countries, and it is the second most frequent cancer and common cause of cancer death among women in countries with low/medium Human Development Index (1). In China, it was estimated that 109,741 new cases of cervical cancer and 59,060 related deaths occurred in 2020, accounting for a significant proportion of the global cervical cancer burden (2). Patients with metastatic or recurrent cervical cancer have poor prognosis and limited treatment options: current standard first-line treatment regimens provide a median progression-free survival (PFS) of <1 year, and most patients who experience disease progression within 1 year would soon need a second-line treatment (3, 4). However, to date, there is no standard of care for advanced cervical cancer for the second-line setting and beyond. The latest Chinese guidelines for the diagnosis and treatment of cervical cancer recommend participation in clinical trials for recurrent and persistent cervical cancer, indicating an unmet need for novel therapies in this setting (5).

Targeting the programmed death-1 (PD-1)/programmed death ligand 1 (PD-L1) axis is a promising treatment strategy for cervical cancer. This is supported by the observation that human papillomavirus, the main causative agent for cervical cancer, increased PD-L1 expression and thereby induced immune evasion in this disease (6, 7). In the second- or later-line setting, single-agent pembrolizumab or nivolumab showed clinical activity in patients with PD-L1–positive cervical cancer in single-arm clinical trials (8–10); based on these results, both checkpoint inhibitors have been included in treatment recommendations (11). Similar results were also obtained with balstilimab when given as a monotherapy (12). Most recently, cemiplimab, a PD-1 antibody, improved survival in recurrent cervical cancer compared with chemotherapy in a phase III study, further confirming the benefit of checkpoint inhibitors in the second-line setting and beyond (13). Nevertheless, tumor responses with these immune checkpoint inhibitors were modest, with objective response rates (ORRs) of <30% (8–10, 12, 13).

Before immune checkpoint inhibitors were investigated in multiple clinical trials, systemic agents such as taxanes and platinum-based chemotherapy were commonly used. Nanoparticle albumin-bound (nab)-paclitaxel is one such chemotherapeutic option, recording the highest ORR (28.6%) when given as a single agent against drug-resistant cervical cancer compared with other chemotherapies (14). Nab-paclitaxel also has antiangiogenic properties similar to those of bevacizumab, and moderate toxicity in recurrent cervical cancer (14). Based on available evidence, adding a chemotherapeutic agent to immune checkpoint inhibitor presents an attractive strategy to improve tumor response as seen in other solid tumors (15).

Serplulimab (formerly HLX10) is a novel humanized immunoglobulin G4 anti–PD-1 monoclonal antibody that has shown antitumor activity and a manageable safety profile in a variety of cancers (16–18). Serplulimab plus chemotherapy significantly improved overall survival (OS) compared with placebo plus chemotherapy in patients with extensive-stage small cell lung cancer in a phase III trial (18). Based on these studies, serplulimab was granted orphan drug designation by the US Food and Drug Administration for the treatment of small cell lung cancer (19); its New Drug Application was accepted by the China National Medical Products Administration (NMPA) for the same indication (20). Serplulimab has been approved by the NMPA for the treatment of advanced microsatellite instability-high solid tumors; the combination regimen of serplulimab, carboplatin, and nab-paclitaxel has been approved for the first-line treatment of unresectable locally advanced or metastatic squamous non-small cell lung cancer (21, 22).

We conducted a phase II trial to assess the efficacy and safety of serplulimab in combination with nab-paclitaxel in patients with advanced cervical cancer who had progressed on or were unable to tolerate first-line standard chemotherapy.

## 2 Materials and methods

### 2.1 Patients

Eligible patients were aged 18–75 years, had histologically or cytologically diagnosed, PD-L1–positive (combined positive score [CPS] ≥1) cervical cancer, and experienced progressive disease or relapse after receiving standard treatment or were intolerant to first-line chemotherapy. Patients must have at least one measurable lesion per the Response Evaluation Criteria in Solid Tumors (RECIST) version 1.1; an Eastern Cooperative Oncology Group (ECOG) performance status score (PS) of 0 or 1; adequate hematologic, renal, and hepatic function; and a life expectancy of ≥12 weeks.

Key exclusion criteria included prior nab-paclitaxel treatment, prior immune checkpoint inhibitor therapy, active malignancies, central nervous system or leptomeningeal metastasis, preparing to or have received an organ or bone marrow transplant, significant cardiovascular disease, and a history of autoimmune disease. The full list of inclusion and exclusion criteria is provided in the **Supplementary Methods**.

The trial was registered with ClinicalTrials.gov (NCT04150575).

### 2.2 Study design and treatment

This was a single-arm, open-label, multicenter, phase II study conducted in China across 11 study sites. All enrolled patients received serplulimab 4.5 mg/kg plus nab-paclitaxel 260 mg/m^2^ once every 3 weeks (21 days). Nab-paclitaxel was given for up to six cycles and serplulimab for up to 2 years (35 dosing cycles) or until disease progression, initiation of new antitumor therapy, death, unacceptable toxicity, withdrawal of informed consent, or other reasons specified in the protocol. Tumor imaging and antitumor efficacy assessment were performed every 6 weeks during the first 48 weeks, then every 12 weeks. All patients were followed up for safety (30 days and 90 days after the last dose of treatment) and survival (every 12 weeks after the end of treatment). The study protocol was approved by the Ethics Committee or review board of all participating sites. The study was conducted in accordance with the local laws and regulations of China, Declaration of Helsinki, and Good Clinical Practice. Any amendment to the study protocol had to be approved by the ethics committee and application registered with local health authorities according to local requirements. All patients provided written informed consent.

### 2.3 Endpoints and assessments

The primary endpoints were safety and ORR assessed by independent radiological review committee (IRRC) based on RECIST version 1.1. Safety consisted of monitoring and documenting all adverse events (AEs) that were evaluated based on the incidence and severity. All AEs were described according to the Medical Dictionary for Regulatory Activities version 23.1 and graded according to the National Cancer Institute Common Terminology Criteria for Adverse Events version 5.0. For any multiple occurrences of the same event, the one with the worst grade was recorded. Adverse event of special interest included infusion-related reactions and immune-related AEs. ORR was defined as the percentage of subjects whose best overall responses were complete response (CR) and partial response (PR).

Secondary endpoints included ORR (assessed by the investigator according to RECIST version 1.1), PFS (assessed by investigator and IRRC according to RECIST version 1.1), 6-month PFS rate, OS, 6-month OS rate, duration of response (DOR), and disease control rate (DCR) defined as proportion of patients with CR, PR, or stable disease (SD). Both CR or PR must be confirmed by tumor evaluation after 28 days and SD lasting ≥42 days.

### 2.4 Statistical analysis

The full analysis set (FAS), defined as patients who received at least one dose of the investigational product, was the primary analysis set for all efficacy and safety analyses. The per-protocol set (PPS) was a subset of the FAS, consisting of all patients without major protocol deviation. The PPS served as support for the FAS. The safety set (SS) was defined as a collection of patients who received at least one dose of the investigational treatment.

Statistical analyses were performed based on the FAS, PPS, and SS using SAS version 9.2 (or later) software (SAS Institute, Cary, NC). There was no hypothesis testing, as this was an open-label, single-arm study. Confidence intervals (CIs) for ORR were estimated using the Exact (Clopper–Pearson) method. OS, PFS, and DOR were estimated using the Kaplan–Meier method. DOR analysis included patients who had achieved objective response. Patient flow through the trial, and demographic and baseline characteristics, were summarized by descriptive statistics.

## 3 Results

### 3.1 Patient characteristics

**Figure 1:**
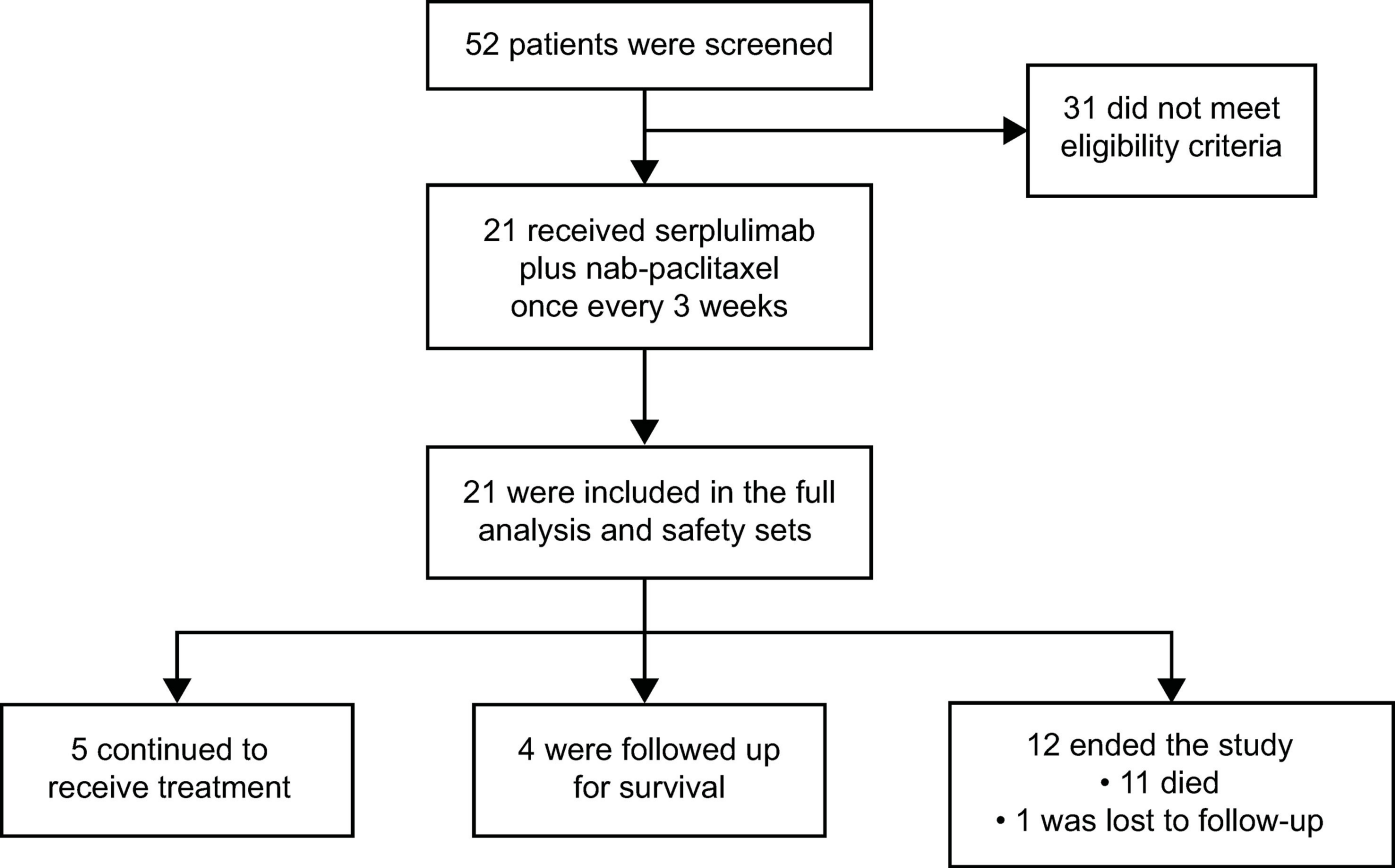
Patient enrollment and disposition.

Between December 27, 2019 and June 30, 2020, of 52 patients screened, 21 were enrolled in the study (**Figure 1**). Thirty-one patients did not meet eligibility criteria, with the most common reasons being CPS <1 (n = 14) and no target lesion (n = 4). As of December 31, 2021, the data cutoff, median follow-up duration was 14.6 months (range, 0.2–21.7). Five (23.8%) patients remained on treatment and 4 (19.0%) were in survival follow-up period. Twelve patients ended the study most commonly due to death (n = 11, 52.4%), while 1 (4.8%) was lost to follow-up.

Patient characteristics and baseline demographics are described in **Table 1**. Mean age at enrollment was 50.8 years (range, 31–64). Most of the patients (71.4%) had an ECOG PS of 1, and almost all (95.2%) had squamous cell carcinoma. Fourteen patients (66.7%) were CPS ≥20; 6 (28.6%) had received two or more lines of antitumor therapy; 18 (85.7%) had received previous radiotherapy; and 9 (42.9%) had lung metastasis at study enrollment.

**Table 1:** Baseline demographics and patient characteristics of the FAS.

Characteristics	Serplulimab + nab-paclitaxel (*N* = 21)
Mean age, years (range)	50.8 (31–64)
Median BMI, kg/m^2^ (range)	26.5 (16.0–28.9)
ECOG PS, *n* (%)	
0	6 (28.6)
1	15 (71.4)
Tumor diagnosis status, *n* (%)	
Local recurrence	7 (33.3)
Distant metastasis	9 (42.9)
Local recurrence and distant metastasis	5 (23.8)
Site of metastasis, *n* (%)	
Lung	9 (42.9)
Distant lymph node	6 (28.6)
Tumor histology, *n* (%)	
Squamous cell carcinoma	20 (95.2)
Adenosquamous carcinoma	1 (4.8)
Combined positive score, *n* (%)	
1≤ CPS <20	7 (33.3)
CPS ≥20	14 (66.7)
Squamous cell carcinoma antigen	
Mean, ng/mL	42.6
Abnormal, *n* (%)	18 (85.7)
Surgery, *n* (%)	12 (57.1)
Radiotherapy, *n* (%)	18 (85.7)
Prior antitumor therapy (≥ second line), *n* (%)	6 (28.6)

BMI, body mass index.

### 3.2 Efficacy

**Figure 2:**
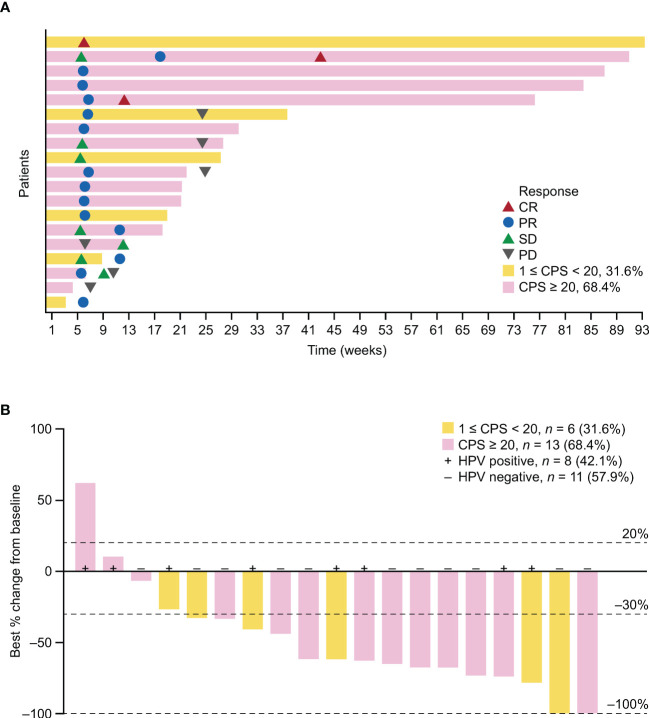
Antitumor activity of serplulimab in the FAS. **(A)** Swimmer plot showing time on treatment, time to best response, and duration of response assessed by IRRC per RECIST version 1.1. **(B)** Best percentage change in target lesion size from baseline. One patient with unconfirmed response was included. PD-L1 status is indicated by color coding of bars. HPV, human papillomavirus; PD, progressive disease.

Objective response was observed in 12 patients and ORR was 57.1% (95% CI 34.0–78.2); 3 (14.3%) patients achieved CR, and 9 (42.9%) achieved PR as their best overall response assessed by IRRC as per RECIST version 1.1 (**Table 2**). Overall, the DCR was 76.2% (95% CI 52.8–91.8). Progressive disease was the best response for 2 (9.5%) patients, and response was non-evaluable for 3 (14.3%) patients. The swimmer plot is presented in **Figure 2A**. The waterfall plot showing distribution of the best percentage change from baseline in target lesion size for the individual patient is shown in **Figure 2B**. Of the 19 patients with at least one post-baseline imaging assessment, 17 (89.5%) had a reduction in target lesion size from baseline and 15 (78.9%) recorded a ≥30% reduction.

**Table 2:** Objective response rate and disease control rate by IRRC.

Tumor response	Serplulimab + nab-paclitaxel (*N* = 21)
CR	3 (14.3)
PR	9 (42.9)
SD	4 (19.0)
PD	2 (9.5)
NE	3 (14.3)
ORR^a^ [95% CI]	12 (57.1) [34.0–78.2]
DCR^b^ [95% CI]	16 (76.2) [52.8–91.8]

All values are shown as n (%).

NE, non-evaluable; PD, progressive disease.

a ORR was defined as the proportion of patients achieving CR or PR.

b DCR was defined as the proportion of patients achieving CR, PR, or SD.

**Figure 3:**
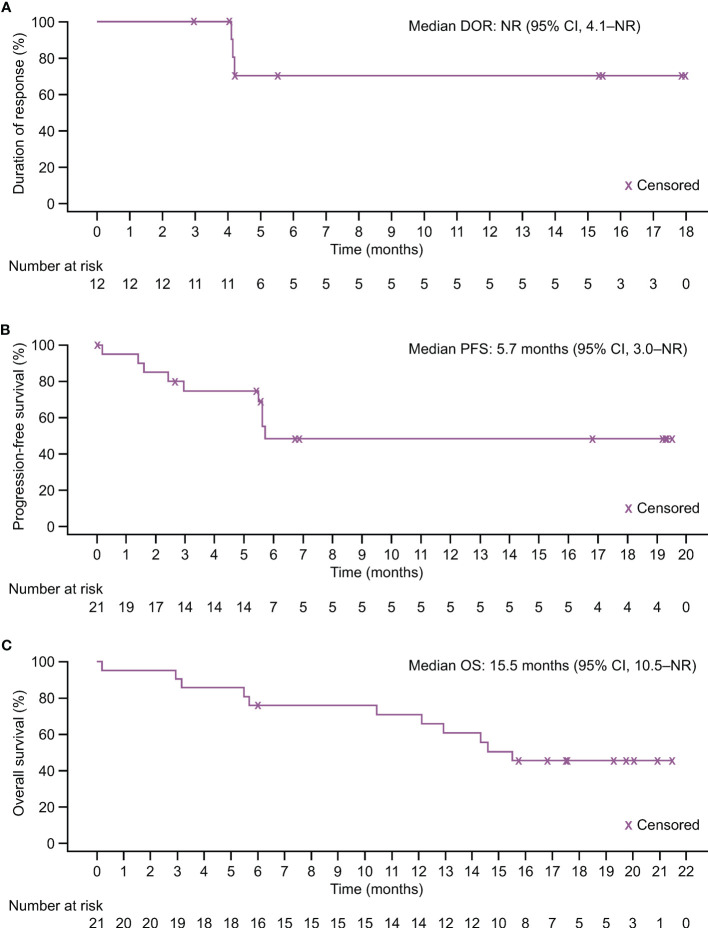
Kaplan–Meier estimates of response duration and survival in the FAS. **(A)** Duration of response assessed by IRRC per RECIST version 1.1. **(B)** Progression-free survival assessed by IRRC per RECIST version 1.1. **(C)** Overall survival.

The median DOR was not reached (NR) (95% CI 4.1–NR months; **Figure 3A**); 12-month DOR rate was 70.0% (95% CI 32.9–89.2). IRRC-assessed median PFS was 5.7 months (95% CI 3.0–NR); both 6- and 12-month PFS rate was 48.2% (95% CI 23.3–69.5; **Figure 3B**). Median OS was 15.5 months (95% CI 10.5–NR); 6- and 12-month OS rates were 76.2% (95% CI 51.9–89.3) and 71.1% (95% CI 46.6–85.9), respectively (**Figure 3C**).

Investigator assessments as per RECIST version 1.1 on the tumor responses are presented in **Supplementary Table 1**. The ORR was 47.6% (95% CI 25.7–70.2) and median PFS was 5.6 months (95% CI 3.0–13.9). None of the patients achieved CR and 10 (47.6%) had PR.

IRCC-assessed ORR stratified by CPS is presented in **Supplementary Table 2**. ORR was 9 (64.3%) among patients with CPS ≥20.

### 3.3 Safety

The most common AEs are summarized in **Table 3**. All patients experienced a treatment-emergent adverse event (TEAE), with 17 (81.0%) of them experiencing grade ≥3 TEAEs. Some of the most common TEAEs were anemia (n =16; 76.2%), white blood cell count decreased (n = 15; 71.4%), neutrophil count decreased (n =14; 66.7%), constipation (n = 11; 52.4%), alopecia (n = 10; 47.6%), and asthenia (n = 9; 42.9%).

**Table 3:** Adverse events of the safety set.

Event	Serplulimab + nab-paclitaxel (*N* = 21)	
	Any grade	Grade ≥3
Any TEAE of incidence >20%		
Anemia	16 (76.2)	5 (23.8)
White blood cell count decreased	15 (71.4)	6 (28.6)
Neutrophil count decreased	14 (66.7)	7 (33.3)
Constipation	11 (52.4)	0
Alopecia	10 (47.6)	0
Asthenia	9 (42.9)	0
Decreased appetite	7 (33.3)	0
Elevated γ-glutamyltransferase	6 (28.6)	3 (14.3)
Weight loss	6 (28.6)	0
Vomiting	6 (28.6)	0
Hypothyroidism	6 (28.6)	0
Nausea	5 (23.8)	0
Pyrexia	5 (23.8)	0
Hypercholesterolemia	5 (23.8)	1 (4.8)
Urinary tract infection	5 (23.8)	0
Any irAE of incidence >10%		
Hypothyroidism	6 (28.6)	0
Hyperthyroidism	3 (14.3)	0
Pruritus	3 (14.3)	0

All values are shown as n (%).

There were no TEAEs leading to serplulimab discontinuation. Four patients died of TEAEs and only 1 who had multiple organ dysfunction syndrome was deemed possibly related to the treatment. Eight (38.1%) patients with serious AEs were reported. Adverse drug reactions (ADRs), defined as events considered to have a causality with the investigational product, were reported in 19 (90.5%) patients. Seven (33.3%) had grade ≥3 ADR. There were no infusion-related reactions.

Immune-related adverse events (irAEs) were observed in 12 (57.1%) patients, including 2 who experienced one or more grade ≥3 event. One of them had elevated blood creatine phosphokinase, while the other had diarrhea and anemia. Both cases were resolved quickly. Hypothyroidism (28.6%), hyperthyroidism (14.3%), and pruritus (14.3%) were the most common irAEs.

## 4 Discussion

The prognosis of recurrent and metastatic cervical cancer is poor and systemic therapy remains the mainstay of treatment. Although various combinations of systemic agents have improved survival outcomes, toxicity remains an issue, and median OS remains modest at around 1 year (23). The addition of bevacizumab to first-line chemotherapy in the landmark GOG 240 study extended median OS to 16.8 months in the bevacizumab chemotherapy arm compared with 13.3 months in the chemotherapy-alone arm; however, there were higher rates of AEs such as genitourinary fistulas, thromboembolic events, and hypertension compared with chemotherapy alone (3, 24). A breakthrough in advanced cervical cancer came in 2021 when pembrolizumab combined with chemotherapy with or without bevacizumab showed improved OS in the recurrent metastatic first-line setting compared with chemotherapy with or without bevacizumab (4). This led to the approval by the US Food and Drug Administration as first-line treatment for PD-L1–positive persistent, recurrent, or metastatic cervical cancer (25).

The efficacy of treatments for patients in the second-line setting and beyond remains low. Systemic chemotherapy was commonly considered, and nab-paclitaxel was one of the more effective options among patients who were resistant to platinum-based chemotherapy. When given as a monotherapy, nab-paclitaxel yielded an ORR of 28.6%, and a PFS and OS of 5.0 and 9.4 months, respectively (14).

The use of immune checkpoint inhibitors in this setting is also an active area of research. In KEYNOTE-158, pembrolizumab yielded an ORR of 12.2% in the overall cohort and 14.6% in those with PD-L1–positive tumors (8). In a more recent update, ORR was 14.3%, and PFS and OS were 2.1 and 9.3 months, respectively, in the overall cohort (9). Another earlier study with nivolumab yielded an ORR of 26.3% in 19 patients with recurrent/metastatic cervical cancer for a median follow-up of 19.2 months (10). More recently, balstilimab attained an ORR of 15% and a median DOR of 15.4 months in 161 previously treated advanced cervical cancer patients (12). Cemiplimab, another anti–PD-1 agent, showed positive clinical activity in a phase III study, reducing the risk of death by 31% compared with chemotherapy and achieving an ORR of 16.4% (13). Although these studies demonstrated the value of immune checkpoint inhibitors in the second-line setting, their clinical activity remained modest. Hence, there is an unmet need for more effective treatment strategies.

The present study has provided evidence that combining immune checkpoint inhibitor with chemotherapy is a promising therapeutic strategy. Serplulimab plus nab-paclitaxel yielded promising antitumor activity, with 5 (23.8%) of 21 patients continuing with treatment at the time of data cutoff. The primary endpoint of ORR assessed by IRRC was 57.1%, and 3 (14.3%) patients experienced CR. Durable clinical activity was observed: median DOR has not been reached after a median follow-up of 14.6 months. The favorable ORR and DOR are further supported by the encouraging median PFS and OS data. Although cross-trial comparisons are difficult and must be interpreted with caution, the ORR in this study was numerically higher than those reported with other immune checkpoint inhibitors, indicating the clinical potential of such treatment combinations. In addition, the ORR was highest at 64.3% among patients with CPS ≥20. Of the 5 patients who had the longest DOR, 4 were CPS >50, suggesting that CPS was positively associated with the efficacy of this treatment combination.

No new safety signals were observed in our study, with most common TEAEs such as anemia, elevated γ-glutamyltransferase, and incidence of irAEs consistent with previous reports (16). Cytotoxic AEs such as lower neutrophil and white blood cell count were reported, which was not unexpected given that nab-paclitaxel was added to the treatment regimen. Most of the TEAEs and irAEs were grade 1 and 2, and the irAEs observed in this study were typical of other immune checkpoint inhibitors (4, 8, 12). Furthermore, there were no infusion-related reactions reported with serplulimab administration or discontinuation of the immune checkpoint inhibitor due to TEAEs. Taken together, serplulimab plus nab-paclitaxel was well tolerated with a manageable safety profile.

To our knowledge, this is the first published study using an immune checkpoint inhibitor plus chemotherapy in the second-line setting for advanced cervical cancer. Studies in other cancers have demonstrated that the addition of chemotherapy to PD-1/PD-L1 inhibitor improved ORR and clinical outcomes, with experts theorizing that such combination could exert a synergistic antitumor effect in patients (26, 27). Hence, this study provided evidence and rationale to combine chemotherapy with an immune checkpoint inhibitor in cervical cancer to maximize the clinical efficacy. Another strength of this study is the evaluation of tumor response from both IRRC and the investigator, giving a more accurate assessment of the benefit of serplulimab. Small sample size and absence of a control arm are limitations of our study, which may affect the robustness of the results. Our limited sample size does not enable clear inferences about the benefit of treatment in those with higher PD-L1 expression, which is a valid biomarker in cancer. As such, we could not establish an association between ORR or PFS with CPS scores. Although our study showed relatively high response rates, further confirmatory data from larger randomized trials are needed, to identify potential biomarkers of response and to establish an association between clinical efficacy and PD-L1 expression levels.

In conclusion, serplulimab plus nab-paclitaxel was associated with durable clinical activity in patients with advanced cervical cancer who had progressed on or were unable to tolerate first-line chemotherapy. Together with a manageable safety profile, this combination treatment represents a clinically meaningful option in the second-line setting. A phase II study of serplulimab plus bevacizumab in combination with chemotherapy in the first-line setting is currently planned for patients with recurrent or metastatic cervical cancer (ClinicalTrials.gov identifier: NCT05444374).

## Data availability statement

The original contributions presented in the study are included in the article/**Supplementary Material**. Further inquiries can be directed to the corresponding author.

## Ethics statement

The studies involving human participants were reviewed and approved by Ethics Committee of National Cancer Center/Cancer Hospital, Chinese Academy of Medical Sciences and Peking Union Medical College. The patients/participants provided their written informed consent to participate in this study.

## Author contributions

JA contributed to data collection, analysis, interpretation, and writing the original draft. XL, JW, LZ, RA, KJ, YH, KW, GL, CW, and JY contributed to data collection, analysis, interpretation, and review of the manuscript. XH contributed to conceptualization, project administration, and review of the manuscript. GY and JL contributed to project administration, resources, and review of the manuscript. QW contributed to conceptualization, project administration, supervision, and review of the manuscript. JZ contributed to resources, supervision, and review of the manuscript. LW contributed to conceptualization, data collection, analysis, interpretation, supervision, and review of the manuscript. All authors contributed to the article and approved the submitted version.
