# Supplementary material for: Efficacy and safety of serplulimab plus nab-paclitaxel in previously treated patients with PD-L1–positive advanced cervical cancer: a phase II, single-arm study
Source: Front Immunol. 2023 Apr 21;14:1142256. doi: 10.3389/fimmu.2023.1142256 (PMC10161140; doi:10.3389/fimmu.2023.1142256)
Supplement: Supplementary file 1 [file DataSheet_1.docx]

Supplementary Material

# Supplementary Methods

## Inclusion criteria

1. Voluntarily participate in, fully understand and be informed about the study, and have signed the informed consent form (ICF); be willing to follow and be able to complete all trial procedures with good compliance.
2. Aged 18–75 years at the time of signing the ICF.
3. Histologically or cytologically diagnosed with cervical cancer.
4. Patients who have experienced progressive disease or relapse after receiving standard treatment (first-line chemotherapy must be included) or who are intolerant to first-line chemotherapy. First-line chemotherapy includes any of the following:
   1. Platinum-based drugs + taxanes;
   2. Platinum-based drugs + topotecan;
   3. Taxanes + topotecan.
5. The radiological examination during screening confirms the presence of at least one measurable lesion evaluated according to the Response Evaluation Criteria in Solid Tumors (RECIST) version 1.1 (central radiographic assessment), which has not received any local treatment including radiotherapy (a lesion located in an area subjected to a previous radiotherapy can be selected as the target lesion if progressive disease is confirmed).
6. Subjects whose tumor specimens are tested positive for PD-L1 expression (combined positive score ≥1); tumor tissues must be collected from these subjects during screening for assay of PD-L1 expression level.
7. Prior systemic antineoplastic therapy must have been ≥2 weeks from the first dose in this study with treatment-related adverse event resolved to NCI-CTCAE grade ≤1 (except for alopecia).
8. An Eastern Cooperative Oncology Group performance status score of 0 or 1 within 7 days prior to the first dose.
9. An expected survival period of ≥12 weeks.
10. Negative hepatitis B surface antigen (HBsAg) and hepatitis B core antibody (HBcAb). In case of positive HBsAg or HBcAb, hepatitis B virus deoxyribonucleic acid must be <2500 copies/mL or 500 IU/mL to be enrolled.
11. Subjects who tested negative for HCV antibody or HCV-RNA are allowed to be enrolled; if HCV-RNA is positive, the subject must have alanine transaminase and aspartate transaminase of ≤3 × upper limit of normal to be enrolled. Patients with coinfection of hepatitis B and C viruses should be excluded.
12. With major organ functions meeting the following criteria within 7 days prior to the first dose (no blood transfusions or treatment with albumin, recombinant human thrombopoietin, or colony-stimulating factor within 14 days prior to the first dose):

| **Hematologic system** | |
| --- | --- |
| Absolute neutrophil count | ≥1.5 × 10^9^/L |
| Platelet | ≥100 × 10^9^/L |
| Hemoglobin | ≥90 g/L |
| **Liver function** | |
| Total bilirubin | ≤1.5 × upper limit of normal (ULN) |
| Alanine transaminase | ≤2.5 × ULN  ≤3 × ULN for HCV-RNA positive  ≤5 × ULN for patients with liver metastases |
| Aspartate transaminase | ≤2.5 × ULN  ≤3 × ULN for HCV-RNA positive  ≤5 × ULN for patients with liver metastases |
| Albumin | ≥30 g/L |
| **Renal function** | |
| Blood creatinine | ≤1.5 × ULN  In case of >1.5 × ULN, creatinine clearance must be ≥60 mL/min  (Calculated using Cockcroft–Gault formula) |
| **Coagulation function** | |
| Activated partial thromboplastin time | ≤1.5 × ULN |
| Prothrombin time | ≤1.5 × ULN |
| International normalized ratio | ≤1.5 × ULN |

1. Women of childbearing age must have a serum pregnancy test within 7 days prior to the first dose and have a negative result. The subjects are required to take a medically approved contraceptive measure (e.g., intrauterine device, contraceptive agent, or condom) during the treatment period, within at least 3 months after the last dose of serplulimab, and within at least 6 months after the last dose of chemotherapy.

## Exclusion criteria

1. Patients who have previously received albumin-bound paclitaxel.
2. Patients with other active malignancies within 5 years or at the same time. Localized tumors that have been cured such as basal cell carcinoma, squamous-cell skin cancer, superficial bladder carcinoma, and breast cancer *in situ* are acceptable.
3. Patients who are preparing for or have received an organ or bone marrow transplant.
4. Presence of uncontrollable pleural effusion, pericardial effusion, or ascites requiring repeated drainage.
5. Central nervous system or leptomeningeal metastases confirmed by imaging or pathological examination.
6. With cerebrovascular accident, myocardial infarction, unstable angina, poorly controlled arrhythmia (including QTc intervals ≥470 ms) (QTc intervals are calculated by Fridericia formula) within 6 months prior to the first dose.
7. Class III to IV cardiac insufficiency according to New York Heart Association classification, or a left ventricular ejection fraction <50% by cardiac color Doppler.
8. With human immunodeficiency virus infection.
9. With active pulmonary tuberculosis.
10. With previous or current interstitial pneumonia, pneumoconiosis, radiation pneumonitis, drug-related pneumonitis, or severely impaired pulmonary function that may interfere with the detection and management of suspected drug-related pulmonary toxicity.
11. With known active or suspected autoimmune diseases. Patients in a stable state with no need for systemic immunosuppressant therapy are allowed to be enrolled.
12. Subjects who have received treatment with live vaccines or live attenuated vaccines within 28 days prior to the first dose; but inactivated viral vaccines for seasonal influenza are allowed.
13. Patients who require systemic treatment with corticosteroids (>10 mg/day prednisone or equivalent) or other immunosuppressive agents within 14 days prior to the first dose or during the study. In the absence of active autoimmune disease, patients are allowed to use inhaled or topical corticosteroids, or adrenal cortical hormone replacement therapy at an effective dose equivalent to ≤10 mg/day prednisone.
14. With any active infection requiring systemic anti-infective therapy within 14 days prior to the first dose.
15. Have received any major surgery (defined as surgeries requiring ≥3 weeks of recovery to be able to receive treatment in this study) within 28 days prior to the first dose.
16. Patients who have received radiotherapy within 28 days prior to the first dose, with the exception of palliative radiotherapy to bone lesions within 7 days prior to the first dose.
17. Patients whose adverse event caused by radiotherapy has not recovered to grade ≤1 (NCI-CTCAE v5.0).
18. Have received any T-cell co-stimulatory or immune checkpoint therapy, including but not limited to cytotoxic T-lymphocyte–associated antigen-4 inhibitors, pd-1 inhibitors, pd-l1/2 inhibitors, or other agents that target T cells.
19. Be in any other ongoing clinical study, or the end of the previous clinical study treatment is <2 weeks from the planned start of this study.
20. With a history of severe allergy to any excipient of study drugs, any monoclonal antibody, and any taxane.
21. Pregnant or lactating women.
22. With any of the following conditions within 6 months prior to the first dose of the study drug:
    1. Gastrointestinal perforation, abdominal fistula, or intra-abdominal abscess;
    2. With bowel obstruction and/or have had clinical signs or symptoms of gastrointestinal obstruction, including incomplete obstruction related to pre-existing conditions or requiring regular parenteral nutrition or tube feeding. Patients with signs/symptoms of incomplete obstruction/obstructive syndrome/bowel obstruction at initial diagnosis can be enrolled in the study if they have received definitive (surgical) treatment to resolve symptoms, such as resection of obstructive lesions;
    3. Intra-abdominal active inflammation, including but not limited to peptic ulcer, diverticulitis, or colitis.
23. With evidence of intra-abdominal gas that cannot be attributed to puncture or recent surgical procedure.
24. With a known history of psychotropics abuse, drug abuse, or alcohol abuse. Patients with a history of alcohol abuse can be enrolled if they have stopped drinking.
25. Have other conditions not suitable for participating in this clinical study as judged by the investigator.

# Supplementary Tables

Supplementary Table 1. Objective response rate, disease control rate, and progression-free survival assessed by the investigator per RECIST version 1.1

| **Efficacy endpoint** | **Serplulimab + nab-paclitaxel**  **(*N* = 21)** |
| --- | --- |
| ORR, *n* (%)^a^ [95% CI] | 10 (47.6) [25.7–70.2] |
| DCR, *n* (%)^b^ [95% CI] | 16 (76.2) [52.8–91.8] |
| CR, *n* (%) | 0 (0.0) |
| PR, *n* (%) | 10 (47.6) |
| SD, *n* (%) | 6 (28.6) |
| PD, *n* (%) | 2 (9.5) |
| NE, *n* (%) | 3 (14.3) |
| PFS, months (95% CI) | 5.6 (3.0–13.9) |
| 6-month PFS rate, % (95% CI) | 37.3 (16.8–58.0) |
| 12-month PFS rate, % (95% CI) | 31.1 (12.3–52.2) |

Abbreviations: CI, confidence interval; CR, complete response; DCR, disease control rate; NE, non-evaluable; ORR, objective response rate; PD, progressive disease; PFS, progression-free survival; PR, partial response; SD, stable disease.

^a^ORR was defined as the proportion of patients achieving CR or PR.

^b^DCR was defined as the proportion of patients achieving CR, PR, or SD.

Supplementary Table 2. IRRC-assessed objective response rates stratified by combined positive score

| **CPS** | **N** | **ORR, *n* (%)^a^** |
| --- | --- | --- |
| 1≤ CPS <10 | 5 | 2 (40.0) |
| 10≤ CPS <20 | 2 | 1 (50.0) |
| CPS ≥20 | 14 | 9 (64.3) |

Abbreviations: CPS, combined positive score; IRRC, independent radiological review committee; ORR, objective response rate.

^a^ORR was defined as the proportion of patients achieving complete or partial response.
